# Gene Expression Pattern and Regulatory Network of *α*-Toxin Treatment in *Bombyx mori*

**DOI:** 10.1155/2019/7859121

**Published:** 2019-03-05

**Authors:** Tieshan Feng, Ping Lin, Jiao Gong, Dong Cheng, Xi Yang, Quan Zhang, Tingcai Cheng

**Affiliations:** ^1^State Key Laboratory of Silkworm Genome Biology, Southwest University, Chongqing 400716, China; ^2^Chongqing Engineering and Technology Research Center for Novel Silk Materials, Southwest University, Chongqing 400715, China; ^3^Chongqing Key Laboratory of Sericultural Science, Southwest University, Chongqing 400715, China

## Abstract

*Bacillus bombyseptieus* is a pathogen of *Bombyx mori*; it can cause bacterial septicemia in silkworm. One of the components of the parasporal crystal toxin of *B. bombyseptieus*, *α*-toxin, plays an important role in the process of infection in silkworm. In this study, we investigated the immune response of silkworm induced by *α*-toxin by using RNA-seq. We compared the changes in gene expression in the midgut, fatbody, and hemocytes of silkworm and in the *B. mori* embryonic cell line (BmE) after treatment with *α*-toxin and identified 952 differentially expressed genes and 353 differentially expressed long noncoding RNAs (lncRNAs). These regulated genes in different tissues were found to be enriched in different pathways. The upregulated genes in the midgut were mainly involved in peptidoglycan catabolic process and tyrosine kinase signaling pathway, whereas the downregulated genes were mainly involved in chitin metabolic pathways. The upregulated genes in fatbody were also involved in peptidoglycan catabolic process, but they were for a different peptidoglycan subtype. Further, genes encoding cecropins were enriched in the fatbody. The downregulated genes were mainly involved in the metabolic pathways of fundamental substances such as cellular protein metabolic process and nucleobase-containing compound metabolic process. These results suggest that *α*-toxin can induce various immune responses in silkworm, and further studies are warranted to understand the mechanism of *α*-toxin action in silkworm. Further, lncRNAs and differentially expressed genes were correlated using coexpression network analysis. Our findings revealed potential candidate genes and lncRNAs that might play important physiological functions in the immune response to *α*-toxins in silkworm.

## 1. Introduction

*Bombyx mori* is a typical model of Lepidoptera. It has been raised for millenarian in China and has gradually spread to Korea, Japan, India, and the West; *B. mori* has a remarkable influence on China's cultural development. In China, sericulture is an important part of husbandry, which is economically beneficial for farmers, as well as accelerates the development of textile, chemical, and pharmaceutical industries. However, a wide variety of pathogens can infect silkworm, even under artificial rearing condition, leading to silkworm death; this remarkably impedes the development of sericulture and results in huge economic losses. Therefore, numerous studies have been focusing on exploring the interaction mechanisms between pathogens and hosts to not only understand the mechanisms of silkworm infections, but also the physiology of silkworm parasites and their hosts as well as other Lepidoptera insects.

Pathogenic microorganisms infecting silkworm include microsporidia [1], nucleopolyhedrovirus (BmNPV) [2], and *Bacillus bombyseptieus* [3]. *B. bombyseptieus* is the leading cause of black chest septicemia. Under high temperature and humidity conditions, it can cause the death of silkworm within three days. Bacteria have evolved many strategies to successfully invade the host, one of which is the secretion of toxins. Bacterial toxins can cause numerous severe immune responses and even lead to the death of the hosts. Some bacterial toxins such as pore-forming toxins form pores in the membranes of bacteria, plants, and mammals, causing membrane permeability and tissue damage [4]. Therefore, the potential applications of toxin research include the development of novel biological insecticides and generation of insect-resistant plants by using transgenic technology. *B. bombyseptieus* can also produce parasporal crystal (PC) toxin that is critical for its invasion. The PC toxin shows oral pathogenic activity and lethality toward silkworms and thus can be used for integrated pest management [5]. As insect pests develop resistance to chemical insecticides, PC toxin is a promising biological insecticide with a range of benefits. Further, studying the immune responses of silkworm to bacterial toxins can contribute to further insights into the interaction between hosts and pathogens [6, 7]. One of the constituents of PC toxin, *α*-toxin, shows oral lethality to silkworm larvae and promotes infection and disease. The mechanism of pathogenesis caused by *α*-toxin needs to be understood. Previous studies have explored the molecular mechanism of *α*-toxin action and showed that it can interact with BmGRK2, leading to host death [8]. However, some problems regarding the immune responses of silkworm induced by *α*-toxin still need to be clarified. Different methods have been used to elucidate the immune response of silkworm after *B. bombyseptieus* infection. The qRT-PCR analysis showed that the Toll pathway is slightly activated [9]. Microarray technology revealed the upregulation of antimicroorganism peptides [10] and the differential expression of some genes [11]. To determine whether different tissues challenged by *α*-toxin have different responses according to the kinds of functionalities, we surveyed the genome-wide transcriptional response and revealed candidate genes and long noncoding RNAs (lncRNAs) involved in the immune response of the midgut, fatbody, and hemocytes of silkworm and in the *B. mori* embryonic cell line (BmE) treated with *α*-toxin by using a high-throughput RNA-seq method. The results might help in understanding the interaction patterns of pathogens and hosts, providing further theoretical basis for the usage of *α*-toxins.

## 2. Materials and Methods

### 2.1. Experimental Insects

The silkworm strain Dazao was used in this study; it was reared at a stable temperature of 25°C. Three-day-old fifth instar larvae after 6 h of starvation were used for the infection experiments. Mulberry leaves were cut into 1 cm^2^ pieces and 50 *μ*g of *α*-toxin. A silkworm and treated mulberry leaf were placed in a petri dish until the mulberry leaf was consumed. After 24 h, triplicates of treated and normal samples of fatbody, midgut, and hemocytes were obtained [5, 12].

### 2.2. RNA Extraction, Library Preparation, and Sequencing

The total RNA was extracted using TRIzol (Invitrogen, USA), according to manufacturer's instructions. 24 individual whole transcriptome strand-specific total RNA libraries were constructed from the control (CK) and *α*-toxin-treated (toxin) samples. All libraries were sequenced using an Illumina HiSeq 2000 machine with a read length of 2 × 100 nt. The data were deposited in the National Center for Biotechnology Information (NCBI) Short Read Archive (SRA) database under the accession number PRJNA472176.

### 2.3. Data Analysis

For quality control, adapter sequences were trimmed, and low-quality bases were filtered from both ends; reads shorter than 50 bp were discarded. Clean reads were obtained using Trimmomatic version 0.35. We corrected sequencing errors by using Rcorrector [13]. To remove reads from rRNAs, we aligned the reads against silkworm rRNA gene sequences downloaded from the silkworm genome database [14] by using Bowtie2 [15] version 2.2.6 with default parameters and removed any matching reads. Next, we aligned the resulting reads to the silkworm genome downloaded from the SilkDB by using HISAT2 [16] version 2.0.5 and SAMtools version 1.3.1 [17]. To maximize the usage of splice junction sites identified from all data, we adopted the previously described two rounds of mapping strategy [18]. We relocated the multimapped reads by using MMR [19] and assigned each read to a unique mapping location.

### 2.4. Differential Expression Analysis of Genes and lncRNAs

The expression level of genes downloaded from SilkDB was calculated as transcripts per million (TPM) [20] by using StringTie [21] version 1.2.3, and the scaling was normalized as trimmed mean of *M* values (TMM) [22] by using edgeR version 3.12.1 [23]. Differential expression analysis was performed using edgeR. Genes with a false discovery rate (FDR) of less than 0.05 and a logFC of more than 1 were considered as differentially expressed [24]. The intersection of upregulated genes was plotted as a matrix layout by using UpSet package [25]. To identify gene clusters, we performed hierarchical cluster analysis based on the Euclidean distance of differentially expressed genes (DEGs) and determined gene clusters by using R package dynamicTreeCut version 1.63-1. To functionally analyze all DEGs, we performed Gene Ontology (GO) [26] enrichment analyses by using GOseq [27] version 1.22.0. For the quantification of lncRNAs, the clean reads were aligned to the lncRNA sequences downloaded from the *B. mori* noncoding RNA database (BmncRNAdb) [28] by using Bowtie2. The expression level for lncRNAs was calculated as TPM by using RSEM [29] version 1.3.0, and the scaling was normalized as TMM by using edgeR. Differential expression analysis was performed using edgeR. lncRNAs with a FDR of less than 0.05 and a logFC of more than 1 were identified as differentially expressed.

### 2.5. Quantitative Real-Time PCR Analysis

Total RNA samples from *α*-toxin-treated and untreated silkworm larvae were extracted using the same method described above. Ten randomly selected DEGs from the fatbody and ten randomly selected lncRNAs from the fatbody and midgut were analyzed using quantitative real-time PCR (qRT-PCR) with three replicates to verify the expression level. The qRT-PCRs were performed using the NovoStart®SYBR qPCR SuperMix (Novoprotein, Shanghai, China) and Real-Time PCR System qTOWER2.0. A final 20 *μ*L qRT-PCR mixture contained 10 *μ*L NovoStart®SYBR qPCR SuperMix, 2 *μ*L diluted cDNA sample, and 200 nM primers. The relative expression levels were calculated using the 2^−ΔΔCT^ method, and the transcription initiation factor 2 gene (*SW22934*) was used as an internal standard. The primers used in qRT-PCR are listed in Supplementary Tables 1 and 2, and cDNA samples from the treated and untreated larvae were synthesized using GoScript™ Reverse Transcription System (Promega) and used for each pair of primers. Statistical analyses were performed using GraphPad Prism 5.0.

### 2.6. Coexpression Analysis

The coexpression network between genes, genes and lncRNAs, and lncRNAs was constructed using WGCNA [30] package in R (3.3.3) as described previously [31], whereas the similarity was measured as biweight midcorrelation coefficients [32]. The network was analyzed, and cliques were identified using R package igraph version 1.1.2.

## 3. Results and Discussion

### 3.1. Overview of RNA-seq Data

About 157 Gb data were obtained, and over 84% data ultimately remained after quality control. Approximately 27 million paired-end sequencing reads were obtained from each sample (four each from treated and untreated), ranging from 23.6 to 31.3 million reads (Supplementary Table 3). By using the HISAT2 method, we mapped these reads to the reference genome. Except data from the BmE cell line, which had a mapping rate of about 77%, the ratio of mapped reads was above 85%, confirming the quality of the reads.

**Figure 1:**
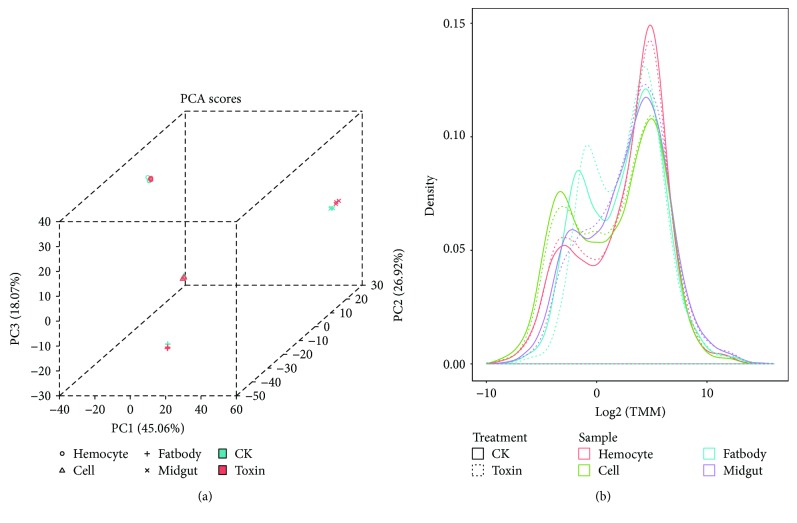
Gene expression profile in response to *α*-toxin challenge. The expression levels are measured as trimmed mean of *M* values transformed by log2. (a) The three-dimensional scatterplot of PCA scores. (b) Kernel density plot of the expression profiles of genes identified as expressed.

After alignment, 8796 genes had read counts of above 5 at least in one sample. To determine whether *α*-toxin treatment affected silkworm growth and whether a difference existed in the response of different tissues, we analyzed the expression of genes obtained from all samples by using principal component analysis (PCA). Replicates were found to cluster and were clearly distinguished from those of other samples, confirming that the distinct difference of transcriptomes among organisms can be detected using RNA-seq. Thus, further analyses could possibly consider the differences among organisms to determine traits correlated with *α*-toxin treatment. The gene expression profiles after *α*-toxin treatment were obviously changed in the midgut and fatbody (Figure 1(a)). To recognize the gene expression pattern, we obtained the kernel density estimates of genes identified as expressed. The distribution of gene expression showed two peaks, which are most of the genes had a high expression level of 32 TMM and some genes had a lower expression level. In different tissues, the expression levels of genes with higher TMM were similar, whereas those of genes with lower TMM value were significantly different. Within the same tissues, the expression levels of genes before and after *α*-toxin treatment were also different (Figure 1(b)).

### 3.2. Differentially Expressed Genes

**Figure 2:**
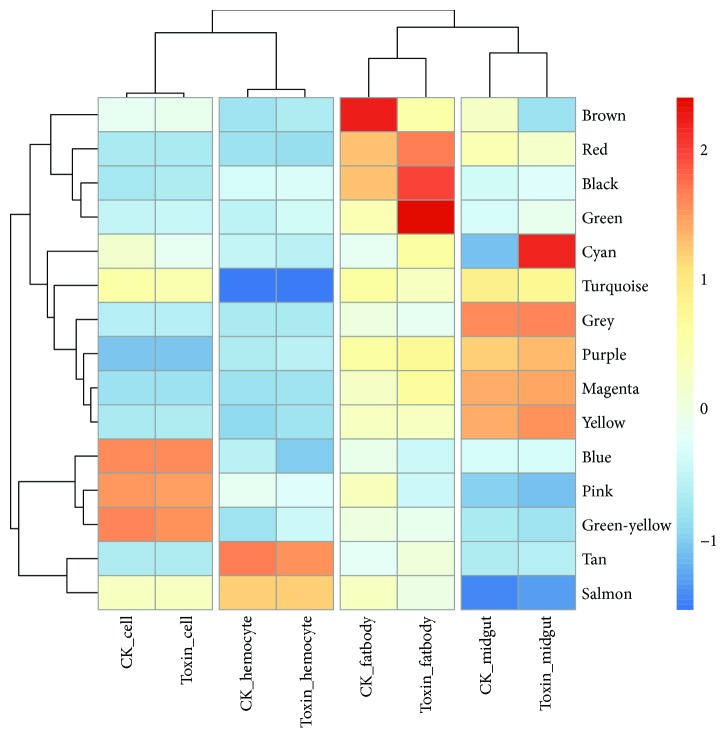
Heatmap plot of clustering result.

In order to understand the effect of *α*-toxin challenge on gene regulation, we identified DEGs by performing differential expression analysis on treated and untreated samples. The number of DEGs in hemocytes, BmE cell line, fatbody, and midgut were 295, 32, 555, and 238, respectively (Supplementary Table 4). To recognize the expression tendency among samples and simplify relevant results, we used hierarchical cluster analysis to group the DEGs into 15 clusters. PCA was performed for each subcluster, and the expression pattern of genes in each subcluster was represented by PC1. Overall, the clusters of all DEGs showed tissue-specific patterns (Figure 2). Among the clusters, those represented in blue, pink, and green-yellow have a high level of expression in the BmE cell line. The genes that showed significant upregulation in these modules were histidine-rich membrane protein KE4, 40S ribosomal protein S21, and arginine kinase, which had a logFC of 8.02, 7.49, and 7.11, respectively. These three proteins are associated with material metabolism and protein synthesis.

Clusters tan and salmon represented a high expression level in hemocyte samples. The genes that were significantly upregulated were Rab11 family-interacting protein 4A and Rab-related protein, which have a logFC of 1.52 and 1.50, respectively. Rab11 family-interacting protein 4A acts as a regulator of endocytic traffic by participating in membrane delivery. In the case of infection by human cytomegalovirus, it might participate in the expulsion of the virus out of the nucleus. Rab proteins are essential for proper organelle functioning; any deviation in the Rab protein cycle leads to physiological disease states [33].

Clusters brown, red, black, and green represent a high expression level in the fatbody samples. The significantly upregulated genes in these clusters were 30K protein 8, protease, cecropin-B, and cecropin-A, which have a logFC of 9.74, 8.97, 8.27, and 8.24, respectively. 30K protein 8 can inhibit ecdysone-induced apoptosis in the BmE cell line [34]. Proteases regulate several invertebrate defense responses, including hemolymph coagulation, antimicrobial peptide synthesis, and melanization of pathogen surfaces [35]. Cecropins are antimicrobial peptides; they lyse bacterial cell membranes and inhibit proline uptake and cause leaky membranes.

Clusters cyan, turquoise, grey, purple, magenta, and yellow represent a high expression level in the midgut samples. The genes showing significant upregulation were tachykinin and gloverin 4, which have a logFC of 7.60 and 4.09, respectively. Tachykinin contributes to multiple disease processes, including acute and chronic inflammation and pain, fibrosis, affective and addictive disorders, functional disorders of the intestine and urinary bladder, infection, and cancer [36]. Gloverin is an inducible antibacterial insect protein that inhibits the synthesis of vital outer membrane proteins, leading to a permeable outer membrane [37].

Taken together, these findings suggest that remarkable differences exist in the functions of DEGs with significant changes in different tissues. According to the physiological function of different tissues, combined with the expression signature of DEGs, we hypothesized a model to analyze the roles of the screened DEGs in silkworm responding to *α*-toxin treatment. The midgut is the main place for *α*-toxin function; *α*-toxin can cause damage to the midgut cells, and thus, the DEGs in the midgut are mainly related to inflammatory response. Fatbody is the major material synthesis and natural immune site of insects; therefore, most of the DEGs in fatbody are related to antimicrobial substance synthesis, detoxification, and natural immune activation. Although hemocytes are not clearly involved in detoxification and immune processes, most of the DEGs can be used as biomarkers for a disease state.

**Figure 3:**
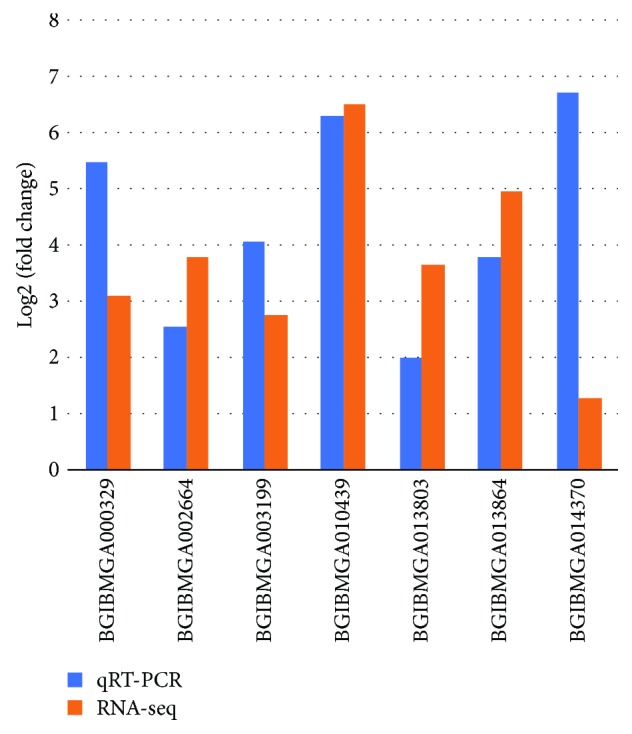
Real-time quantitative PCR results of genes.

To validate the differential expression analysis results, we selected 10 DEGs for qRT-PCR analysis. Seven genes, including gloverin-like protein 3, peptidoglycan recognition protein B, larval cuticle protein, Toll, cuticle protein 3, dopa-decarboxylase, and lysozyme, showed a similar expression pattern as that revealed by RNA-seq (Figure 3). Further, their expression was significantly upregulated (Supplementary Figure 5). This result confirms the credibility of the differential expression analysis results of RNA-seq in this study.

**Figure 4:**
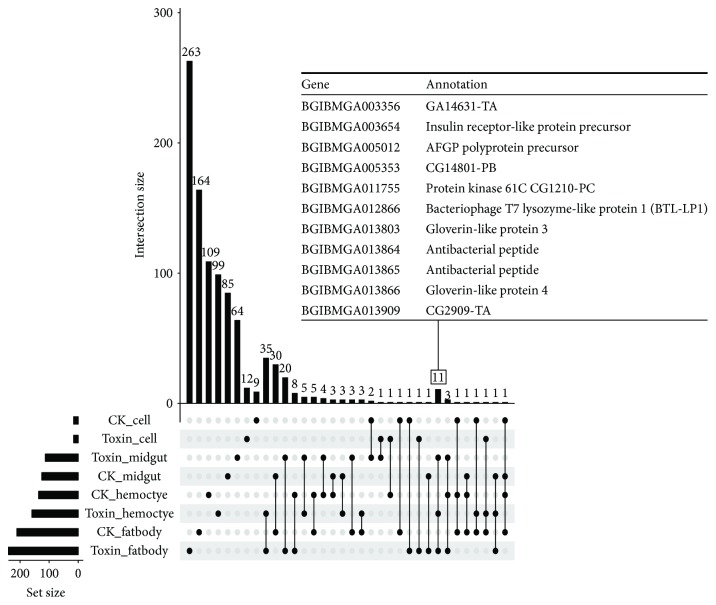
Matrix layout for all intersections of upregulated genes from the four tissues, sorted by size. Dark circles in the matrix indicate sets that are a part of the intersection. The genes common among treated midgut, fatbody, and hemocytes are outlined in the accompanying table.

Among the DEGs, 543 and 441 genes were identified as up- and downregulated at least in one sample, respectively. Less overlapping of DEGs was found among four samples, implying that different tissues have different responses to *α*-toxin (Figure 4). Only eleven genes were commonly upregulated in the treated tissue samples, and several genes encoding antibacterial peptides were upregulated in all the tissues, such as BGIBMGA013866 and BGIBMGA013803, which were annotated as gloverin-like proteins. Gloverin is an inducible antibacterial insect protein that inhibits the growth of bacteria by increasing the permeability of the outer membrane [37, 38]. In particular, BGIBMGA013864 and BGIBMGA013865 were annotated as antibacterial peptides of *B. mori*, indicating that antibacterial peptides might play a common role in different samples in toxin response. A total of 35 genes were upregulated in both treated hemocyte and fatbody samples, including 4 genes annotated as cecropins of *B. mori*. Cecropins are antimicrobial peptides [39] and were first isolated from the hemolymph of *Hyalophora cecropia*. Cecropins lyse bacterial cell membranes and constitute the main part of cell-free immunity of insects [40]. A total of 20 genes were upregulated in both treated fatbody and midgut, including 2 genes annotated as cecropin A of *B. mori* and several genes annotated as heat shock proteins of *B. mori*.

### 3.3. Functional Annotation of DEGs

**Figure 5:**
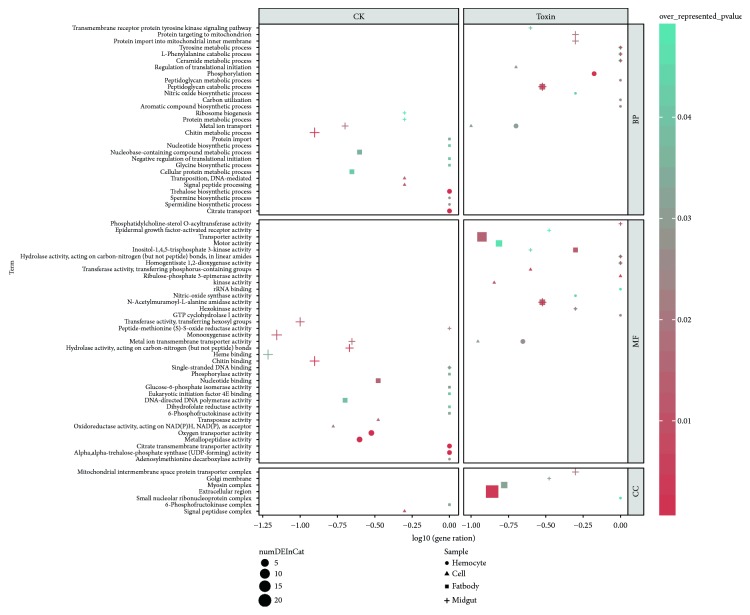
Bubble plot of Gene Ontology (GO) term enrichment results. Gene ratio is calculated as the number of genes in a category divided by the number of genes in the background.

To understand the functions of DEGs, we performed GO analysis. In all, 126 DEGs were divided into two groups (upregulated in the CK or toxin group) and categorized into three functional ontologies (biological processes, cellular components, and molecular functions) and 78 subcategories (Figure 5, Supplementary Table 6). Among these subcategories, genes upregulated in *α*-toxin-treated fatbody, midgut, and hemocytes, including those involved in *N*-acetylmuramoyl-l-alanine amidase activity, GTP cyclohydrolase I activity, metal ion transmembrane transporter activity, nitric-oxide synthase activity, homogentisate 1,2-dioxygenase activity, and hydrolase activity, were enriched in peptidoglycan catabolic process. Genes upregulated in *α*-toxin-treated fatbody and midgut, including BGIBMGA007324 gene, which has the molecular function of homogentisate 1,2-dioxygenase-like, were enriched in tyrosine metabolic process and l-phenylalanine catabolic process. Homogentisate 1,2-dioxygenase is involved in the oxidization of homogentisate and prevents the blackening of tissues, also called melanin. In humans, its deficiency causes a metabolic disease called alkaptonuria [41]. In insects, diverse physiological processes, including wound healing, cuticle tanning, and immunity, are associated with melanization [42]. In addition to these commonly impacted pathways, alterations in distinct biological processes were observed. In *α*-toxin-treated midgut, protein targeting to the mitochondrion, transmembrane receptor protein tyrosine kinase signaling pathway, and protein import into the mitochondrial inner membrane were affected, including several mitochondrial import inner membrane translocase subunits. Translocase of the inner membrane complex is responsible for the translocation of proteins produced from nuclear DNA through the mitochondrial membrane for use in oxidative phosphorylation [43, 44]. In the untreated midgut, protein metabolic process, ribosome biogenesis, and chitin metabolic process were affected, including genes *BGIBMGA007678* and *BGIBMGA007899* annotated as chitinase 3-like, also called YKL-40. The expression of chitinase 3 is associated with the pathogenic process [45]. In *α*-toxin-treated fatbody, several genes annotated as the precursor of cecropins were found to be enriched in the extracellular region of cellular component. In addition to genes encoding antibacterial peptides, other genes associated with immune response and dysfunction of cells were revealed by GO term enrichment analyses. Previous studies have shown that G protein-coupled receptor kinase 2 regulates cAMP/protein kinase A (PKA) signaling pathway to induce host death [8]. Consistent with this finding, our analysis revealed that the upregulated genes in the fatbody were enriched in the molecular function of inositol triphosphate kinase activity associated with the G protein-coupled receptor signaling pathway, including genes *BGIBMGA009298* and *BGIBMGA008451*, which were annotated as inositol 1 and inositol-trisphosphate 3-kinase B-like, respectively. Inositol and some of its polyphosphates function as secondary messengers in many intracellular signal transduction pathways, including gene expression and intracellular calcium concentration control [46–48]. Inositol-trisphosphate 3-kinase plays a vital role in the calcium signaling pathway and is directly regulated by PKA. Calcium can act as a second messenger in the indirect signal transduction pathways such as G protein-coupled receptors signaling pathways. In untreated fatbody, biological process related to the regulation of translation was enriched. The results showed that *α*-toxin affected substance metabolism in the fatbody, which is an important material synthesis organ of silkworm. Treatment with *α*-toxin was found to result in a potentially enhanced immune system, and many immune genes were significantly upregulated. Pathways associated with these genes might be the major signaling pathways affected by *α*-toxin and deserve further investigation.

### 3.4. Differentially Expressed lncRNAs

We performed differential expression analysis of the reference lncRNAs, and a total of 86, 20, 194, and 104 lncRNAs were found to be differentially expressed in the hemocytes, cell, fatbody, and midgut, respectively (Supplementary Table 7). About 42, 11, 87, and 58 lncRNAs were upregulated, and 43, 8, 106, and 45 lncRNAs were downregulated in the hemocytes, cell, fatbody, and midgut, respectively. Among these lncRNAs, two were upregulated in the fatbody, midgut, and hemocytes. In the fatbody and midgut, seven lncRNAs were downregulated, and three were upregulated. In the fatbody and hemocytes, two lncRNAs were upregulated, and five were downregulated.

**Figure 6:**
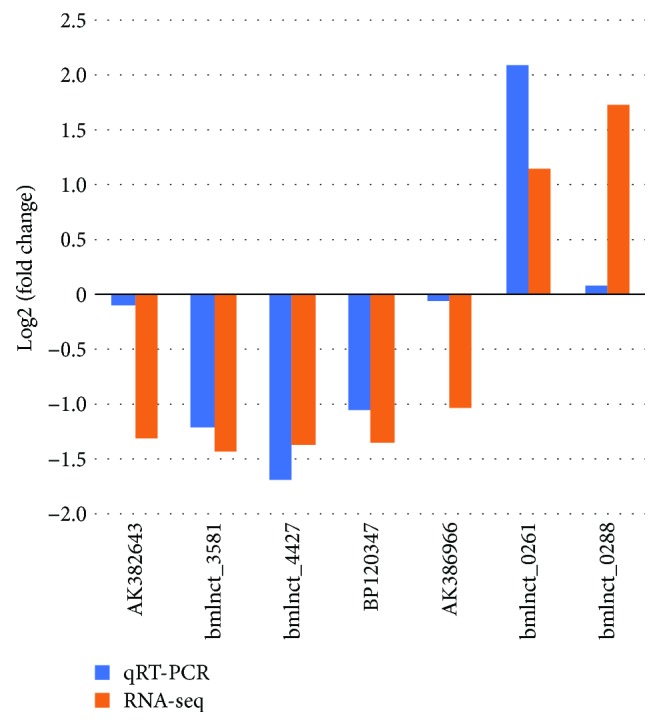
Real-time quantitative PCR results of lncRNAs.

qRT-PCR was performed to verify the expression pattern of nine randomly selected differentially expressed lncRNAs in the fatbody and midgut by using the same cDNAs that were used to verify DEGs. Seven lncRNAs showed the same tendency as that measured using RNA-seq (Figure 6), and 4 lncRNAs (bmlnct_4427, bmlnct_3581, BP120347, and bmlnct_0261) were identified as differentially expressed (Supplementary Figure 8). For the remaining 3 lncRNAs (AK382643, AK386966, and bmlnct_0288), the fold-change values measured using qRT-PCR were not as high as those revealed using RNA-seq. This might be because of the low expression of these lncRNAs or the inefficiency of the qRT-PCR assay to detect the expression level. Therefore, the results of qRT-PCR are generally consistent with those of RNA-seq, particularly for lncRNAs with a high expression level.

**Figure 7:**
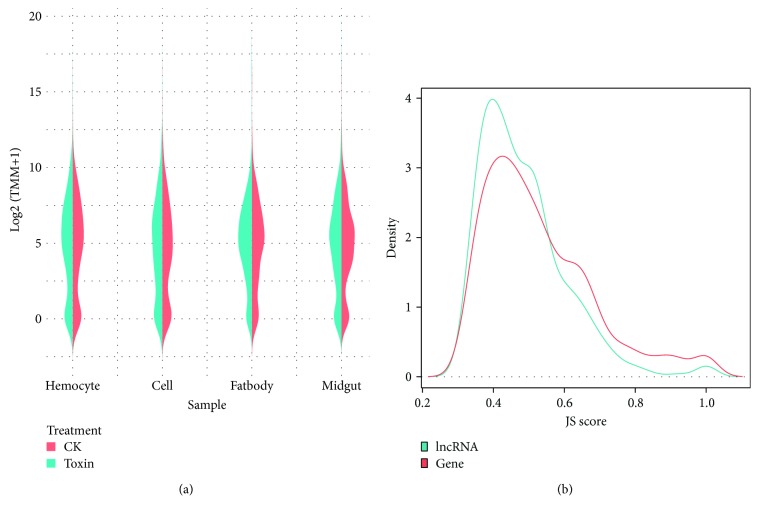
Characteristics of differentially expressed lncRNAs. (a) Violin plot of the expression profile of differentially expressed lncRNAs. (b) Density plot of Jensen-Shannon score of differentially expressed lncRNAs and genes.

The expression of differentially expressed lncRNAs was very similar among all tissues. The density curves of TMM showed double peak distribution characteristics. Most of the differentially expressed lncRNAs had TMM of around 32, and some of them had a lower expression level. The total expression of differentially expressed lncRNAs showed a downward trend after toxin treatment (Figure 7(a)). Compared with the expression distribution of total lncRNAs, the expression distribution of differentially expressed lncRNAs was higher. Previous studies have shown that lncRNAs have stronger tissue specificity than genes [49]. We calculated the Jensen-Shannon (JS) score for the differentially expressed lncRNAs and genes [50] and evaluated their tissue specificity based on the JS score. Most of the differentially expressed lncRNAs had lower JS score than DEGs, indicating that they are more tissue specific than DEGs (Figure 7(b)).

### 3.5. Coexpression Analysis

**Figure 8:**
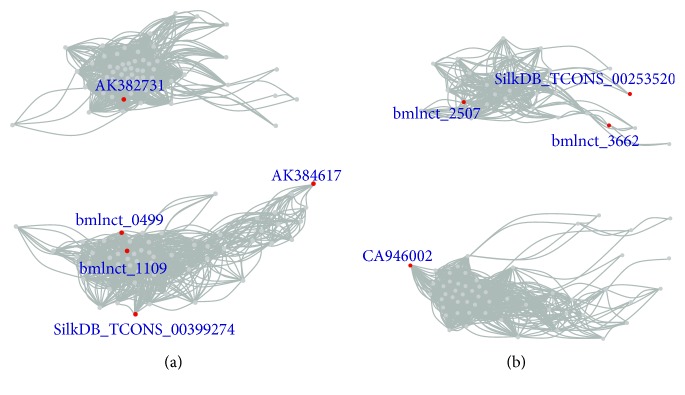
Network visualization. (a) Connections within the blue module. (b) Connections within the red module.

In order to determine whether lncRNAs regulate the immune response of silkworm to *α*-toxin, we constructed a coexpression network of differentially expressed lncRNAs and DEGs. Two modules (blue and red) were identified, including nine lncRNAs and 196 genes. The blue module consists of 118 nodes, including 113 genes and 5 lncRNAs, and the red module consists of 87 nodes, including 83 genes and 4 lncRNAs. To investigate the function of these genes, we separately performed their GO enrichment analysis. Genes of the blue module showed no significantly enriched term. However, the enrichment result of genes of the red module showed that the enriched biological processes were chitin metabolic (*P* value: 0.000163) and transmembrane transport (*P* value: 0.00758), the enriched molecular function was chitin binding (*P* value: 0.000791), and the enriched cellular components were extracellular region (*P* value: 0.000702) and structural constituent of cuticle (*P* value: 0.0224). Comparison among modules revealed 61 genes and two lncRNAs (AK384617 and SilkDB_TCONS_00253520), indicating that different modules are mainly linked by genes. AK384617 is connected with BGIBMGA010913 and BGIBMGA014052, and SilkDB_TCONS_00253520 is connected with BGIBMGA013639 and BGIBMGA008255. Among the four genes, both BGIBMGA008255 and BGIBMGA010913 are annotated as cuticle proteins. The two modules can be divided into two relatively independent small modules. As depicted, the correlation within the modules was more compact than that between modules (Figures 8(a) and 8(b)). To identify the function-related key genes in the network and determine whether lncRNAs are associated with them, we extracted cliques with characteristics of a complete graph from the module (Supplementary Table 9). In the blue module, a total of 82 cliques were identified, and 38 cliques contained lncRNAs. The largest cliques contained 40 nodes, including an lncRNA numbered AK382731. The proteins encoded by the genes associated with this lncRNA included serine proteases and cytochrome P450. Serine proteases are found ubiquitously in different tissues and are responsible for various physiological functions, including immune response [51, 52]. Cytochrome P450 is the major enzyme involved in drug metabolism, and its activity can be affected by many drugs [53]. In the red module, a total of 57 cliques were identified, of which nine contained lncRNAs. Among the nine cliques, five contained an lncRNA numbered bmlnct_2507. The genes associated with bmlnct_2507 include cecropins and cuticular proteins. These lncRNAs represent candidates for further research to determine whether they are important for host-pathogen interactions or bacterial toxin resistance.

## 4. Conclusions

In this study, 952 genes and 353 lncRNAs were identified as differentially expressed among all tissues. Several genes related to immune response and detoxification were upregulated in the fatbody, and qRT-PCR results showed that their expression level was significantly changed after *α*-toxin treatment, indicating that *α*-toxin might trigger defense responses in silkworm. Further, several lncRNAs showed coexpression with the differentially expressed genes, indicating that lncRNAs likely play a potential role in the host response of silkworm.

## Data Availability

The datasets supporting the conclusions of this study are available under BioProject accession number PRJNA472176 of the NCBI BioProject database (https://www.ncbi.nlm.nih.gov/bioproject/).

## Supplementary Materials

Supplementary 1 Table 1: primers used for qRT-PCR for the validation of the selected differentially expressed genes.

Supplementary 2 Table 2: primers used for qRT-PCR for the validation of the selected differentially expressed lncRNAs.

Supplementary 3 Table 3: statistics of the mapped reads for each sequencing library.

Supplementary 4 Table 4: differential expression analysis and cluster analysis results of genes.

Supplementary 5 Figure 5: the qRT-PCR results of genes. ∗ indicates a *t*-test *P* value lower than 0.05, and ∗∗ indicates a *t*-test *P* value lower than 0.01.

Supplementary 6 Table 6: go enrichment analysis for the upregulated and downregulated genes in the fatbody and hemocyte.

Supplementary 7 Table 7: differential expression analysis results of lncRNAs.

Supplementary 8 Figure 8: the qRT-PCR results of lncRNAs. ∗ indicates a *t*-test *P* value lower than 0.05, and ∗∗ indicate a *t*-test *P* value lower than 0.01.

Supplementary 9 Table 9: cliques within the blue and red module.
