# Supplementary figures and images for: Gene Expression Pattern and Regulatory Network of α-Toxin Treatment in Bombyx mori
Source: Int J Genomics. 2019 Mar 5;2019:7859121. doi: 10.1155/2019/7859121 (PMC6425383; doi:10.1155/2019/7859121)

BGIBMGA000329

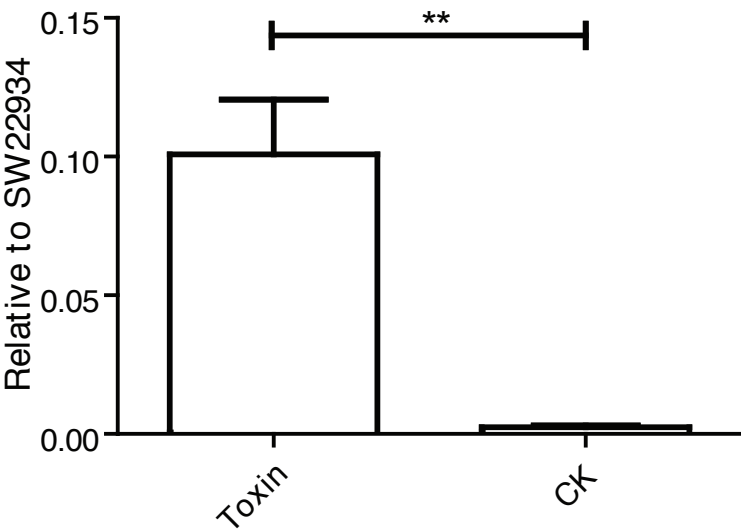

BGIBMGA014370

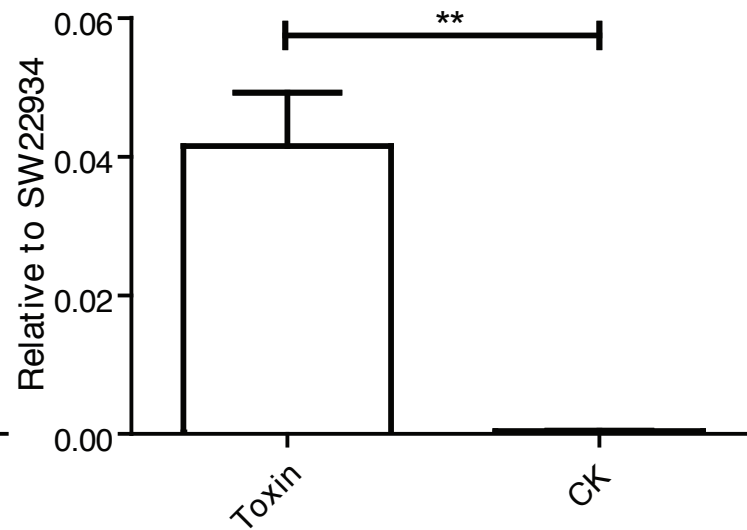

BGIBMGA013864

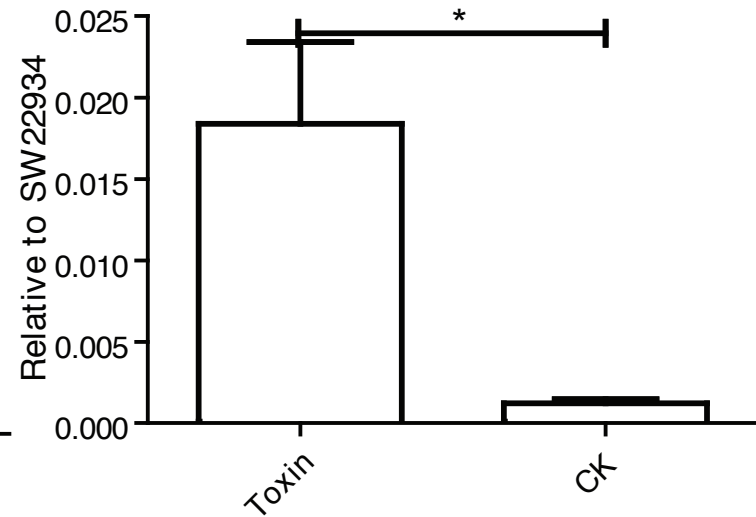

BGIBMGA013803

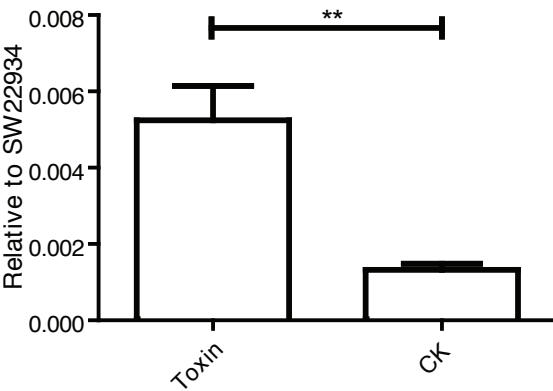

BGIBMGA010439

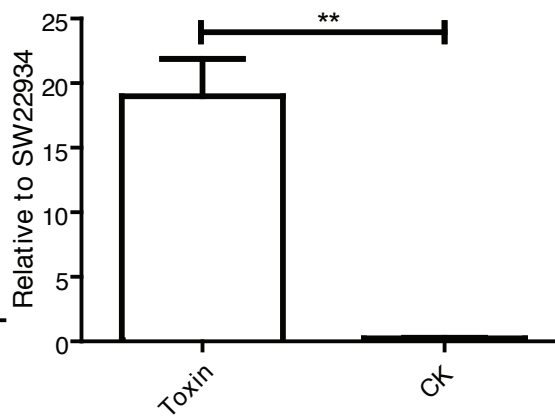

BGIBMGA003199

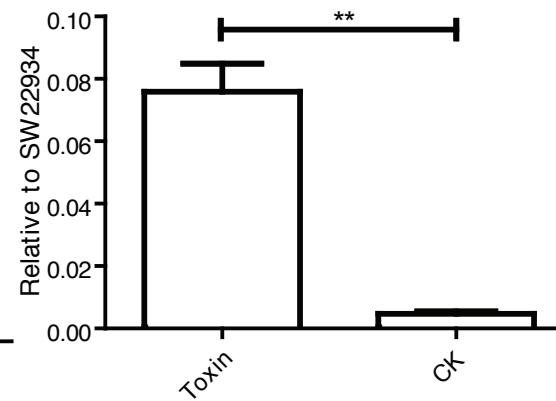

BGIBMGA002664

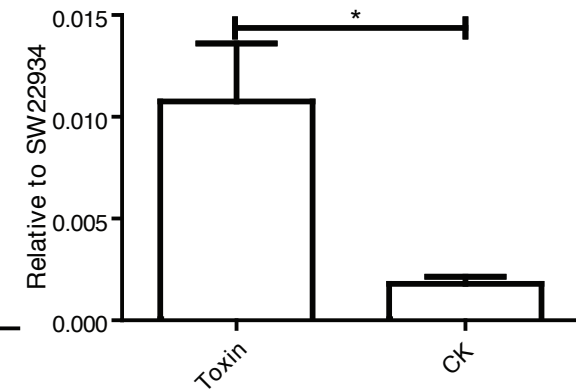

Supplement: Supplementary 5 — Figure 5: the qRT-PCR results of genes. ∗ indicates a t-test P value lower than 0.05, and ∗∗ indicates a t-test P value lower than 0.01. [file 7859121.f5.pdf]

bmlnct\_0261

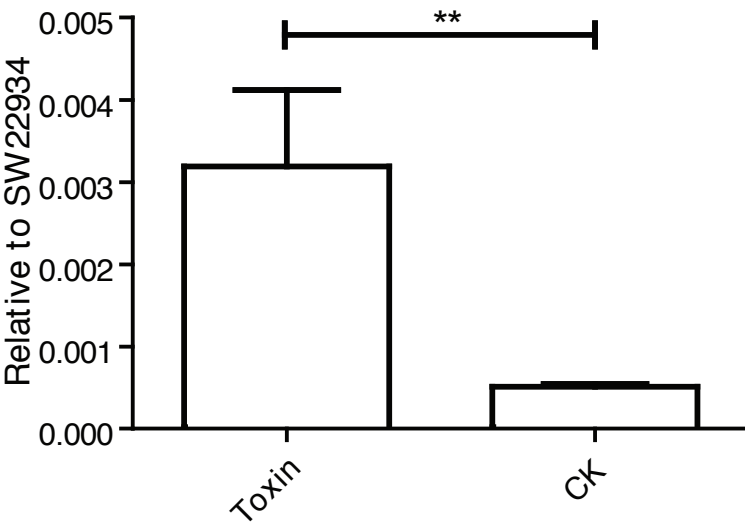

BP120347

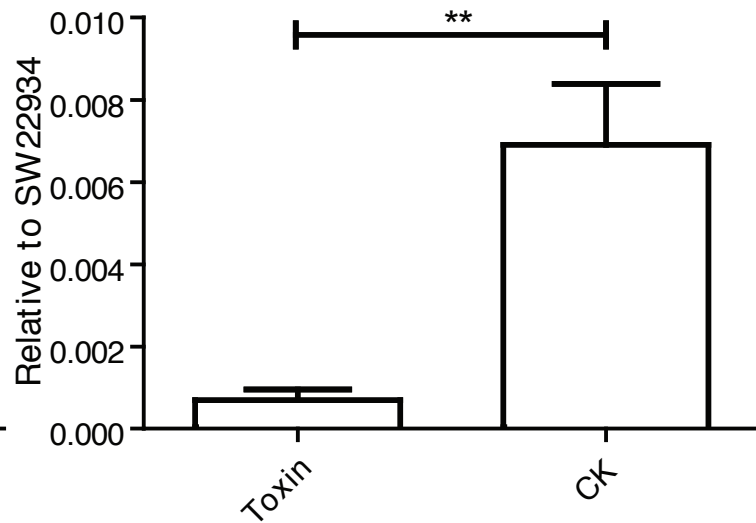

bmlnct\_4427

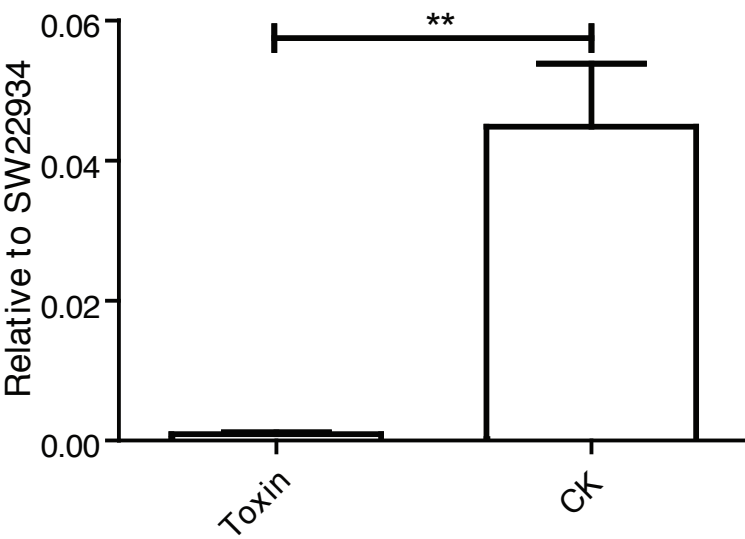

bmlnct\_3581

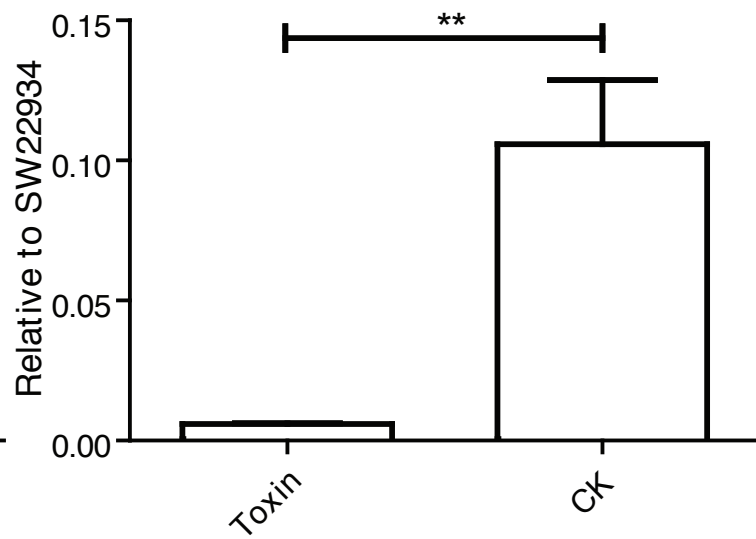

Supplement: Supplementary 8 — Figure 8: the qRT-PCR results of lncRNAs. ∗ indicates a t-test P value lower than 0.05, and ∗∗ indicate a t-test P value lower than 0.01. [file 7859121.f8.pdf]
